# Periodontal status during pregnancy and postpartum

**DOI:** 10.1371/journal.pone.0178234

**Published:** 2017-05-19

**Authors:** Maximino González-Jaranay, Luís Téllez, Antonio Roa-López, Gerardo Gómez-Moreno, Gerardo Moreu

**Affiliations:** Department of Periodontology, Faculty of Dentistry, Granada University, Granada, Spain; Virginia Commonwealth University, UNITED STATES

## Abstract

**Objectives:**

Different studies have documented an association between periodontal disease and low birth-weight delivery. Hence, knowledge of periodontal status during pregnancy and postpartum is important in order to reduce the risks of both diseases. This study aimed to analyze periodontal status at successive stages of pregnancy and 3–6 weeks postpartum in women with initial periodontal alterations.

**Materials and methods:**

Ninety-six pregnant women were examined at 8–10 weeks (pregnancy diagnosis, baseline), 21–23 weeks and 34–36 weeks of gestation and at 40 days postpartum to record plaque scores, clinically assessed gingival inflammation and probing depth (mean depth and % sites with depth >3 mm). Bivariate and multivariate analyses were performed. Type 1 (α) error was established at 0.05

**Results:**

Plaque Index increased (p = 0.043) throughout pregnancy (baseline, 42%±0.18); 21–23 weeks, 42.6%±0.14; 34–36 weeks, 45.6%±0.13 and decreased postpartum (44.8%±0–13). Gingival Index increased (p<0.001) throughout pregnancy (baseline, 56.7%±0.20; 21–23 weeks, 66.36%±0.17; 34–36 weeks, 74.5%±0.18) and decreased postpartum (59.3%±0.21). Probing Depth increased (p<0.001) throughout pregnancy (baseline, 2.51±0.05; 21–23 weeks, 2.63±0.053; 34–36 weeks 2.81±0.055) and decreased postpartum (2.54±0.049). Percentage of sites with Probing Depth >3 mm increased (p<0.001) throughout pregnancy (baseline, 17.6%±0.16; 21–23 weeks, 23.9%±0.17; 34–36 weeks, 31.1%±0.17) and decreased postpartum (21.2%±0.17) but remained significantly (p<0.02) higher than at baseline.

**Conclusion:**

Periodontal status deteriorates during gestation but improves postpartum.

## Introduction

Although the evidence suggests that pregnancy is not in itself a risk factor for periodontal disease, gingival disorders are well documented in the mother during the second and third trimester of gestation [1, 2]. It is therefore important for pregnant women to achieve meticulous plaque control and receive preventive periodontal treatment.

Changes and increases in sexual hormones during pregnancy affect different organs and produce an alteration of the immune system [3–8]. There is an inhibition of T cell activity [9, 10], reduction in chemotaxis and phagocytosis of neutrophils, alteration in lymphocyte response and decrease in antibody production [11–14]. There have also been reports of chronic maternal stress [15] and nutritional deficit associated with the nutritional demands of the mother or fetus [16]. Estrogen and progesterone receptors in the gingival [17] would explain, among other factors, the increased gingival response to plaque during pregnancy [18]. There is evidence that gingival tissues are influenced by physiological changes in serum concentrations of female sex hormones during gestation, producing some degree of gingival oedema and gingivitis in around 50% of pregnant women [19–21]. There is also an increase in gingival capillary permeability and a resulting increase in the flow of crevicular fluid [22]. Moreover, the bacterial composition is itself modified by increased progesterone levels that favour the development of *Prevotella intermedia* [23, 24]. These factors may account for the increased gingivitis during pregnancy, with characteristic interdental tumefaction or even epulis [25]. These symptoms preferentially appear in anterior areas. Finally, at around the 8^th^ month there can be dental hypermobility [26] that then abates, as do the gingival symptoms.

Host inflammatory and immune responses play a major role in periodontal disease. Periodontal disorders are initiated and persist because of factors related to the subgingival microflora. There is an increased presence of microorganisms such as *Porphyromonas gingivalis*, frequently isolated in periodontitis, and in the gingival crevicular fluid of pregnant versus non pregnant women [23, 27]. Furthermore, different studies have documented an association between periodontal disease and low birth-weight delivery [28–30] or poor pregnancy outcome [31]. Study of maternal periodontal status during pregnancy and postpartum can contribute to elucidating this interrelationship and to the design of dental healthcare strategies for pregnant women. In fact, various preventive therapies based on oral hygiene educational programmes have been proposed for application throughout pregnancy to avoid onset of these periodontal disorders [29, 32].

This study aimed to analyze the periodontal status of pregnant women with initial periodontal alterations at successive stages of gestation and postpartum.

## Materials and methods

The study was conducted at a local Health Centre after approval by the Ethics Committee of the Health District and by the Scientific Committee of our School of Dentistry and according to the principles outlined in the Declaration of Helsinki on experimentation involving human subjects. This health centre runs an oral-dental health programme for pregnant women providing regular examinations during and after pregnancy. The study population comprised women undergoing the programme who met the following study inclusion criteria: pregnant women, uniparus or multiparus, with no history or presence of systemic disease, aged between 18–40 years, with normal pregnancy, not current tobacco, alcohol or drug (heroin, cocaine, etc.) users, possessing more than 20 teeth and with some degree of gingivitis or periodontitis, defined as bleeding on probing or probing depth > 3mm at any site. All of the women who attended on one of the two weekdays randomly selected for the cohort formation, a total of 374 women, received an initial examination. The period to form a closed cohort of 100 pregnant women who fulfilled these inclusion criteria was 20 months. The study subjects provided written informed consent to their participation. After four women were excluded after suffering miscarriage, the final study sample comprised 96 women. The initial baseline examination was performed on the date of the pregnancy diagnosis (i.e., during first 8–10 weeks of gestation). The women were followed up, as indicated by the obstetricians, at 21–23 weeks and 34–36 weeks of gestation and at 3–6 weeks postpartum; each of these three measurements were considered different levels of exposure to pregnancy and compared with the initial measurement, considered as reference. At each examination, the general clinical history was reviewed, routine biochemical and haematological parameters were recorded to establish the absence of disease, and oral examination was performed to determine scores on the gingival index of Ainamo & Bay [33] and plaque index of O’Leary [34] and to record probing depths using a PUNC 15 PCR probe. Three study points were measured in each tooth (distal, mid, and mesial) on both bucal and lingual aspects. The mean probing depth of examined sites and the proportion of these with probing depth of >3 mm were recorded for each patient. A single dentist (LTB) performed all examinations.

In order to test the validity of the bleeding and probing depth measurements, previous inter-examiner calibration was performed using Cohen’s Kappa Test. For this purpose, ten patients were randomly selected for gingival and probing depth measurements and examined by two observers.

### Statistical analysis

The periodontal parameters, assessed in the full mouth, were considered as continuous variables: plaque index as % of stained area; gingival index as % of bleeding area; and probing depth as mean of all examined sites and as % of these with > 3 mm depth. The successive measurements of periodontal parameters were studied by constructing a general linear model of repeated measurements for each one, in which the four measurements taken during the study were defined as the intrasubject factor. Significant differences in measurements were determined by single and repeated comparison of repeated measurements, defining the last category as the reference of the variable. The intrasubject effects (periodontal measurements) were compared using the Greenhouse-Geisser statistic, a correction used in univariate repeated measurements when the assumption of sphericity is violated (determined by the Mauchly test).

The statistical analysis was performed using the IBM SPSS Statistics 23.0 statistical package for Windows (Chicago: SPSS Inc.), the SUDAAN 11.0 program (Durham: RTI International) for correlated data (for entering questionnaire responses and clinical data), and the Microsoft Excel 2013 (v15.0) program for Windows (Redmond: Microsoft Corp.) for entering periodontal variables. The type 1 (α) error established for all analyses was 0.05

## Results

A total of 374 pregnant women were recruited, from whom a final study group of 96 women was selected, with an age (mean±standard error) of 29.32±0.45 yrs (range, 18–40 yrs) and weight of 64.18±0.993 Kg (range 39.5–106 Kg). Among the final study group, 89 had a full-term pregnancy and seven miscarried. The gestation period was 39.11±0.19 weeks (range, 34–42 weeks); 36 of the women were primiparus and 60 multiparus.

In the inter-examiner reliability test, the agreement was 86.3% for the gingival index (Kappa = 0.725±0.043; p<0.001) and 81% for the probing depth (Kappa = 0.94; CI [0.9334–0.9458]; p<0.001).

### Plaque index

The measurements obtained at each measurement time in the women who reached full term showed an increase during the pregnancy [42%±0.18 at baseline, 42.6%±0.14 at 21–23 weeks, and 45.6%±0.13 at 34–36 weeks] and a slight recovery [44.8%±0.13] at 3–6 weeks after delivery. Although these changes were not very relevant clinically, the global analysis showed them to be statistically significant (Greenhouse-Geisser: 3.013; d.f. = 2.35; p = 0.043). According to single and repeated multiple intrasubject comparisons, the plaque measurement at 34–36 weeks significantly differed from that at baseline and 21–23 weeks (Table 1).

**Table 1:** Multiple comparisons among the different periodontal measurements.

Periodontal measurements	21–23 weeks vs. baseline		34–36 weeks vs. baseline		3–6 weeks after delivery vs. baseline	
	F*	Significance	F*	Significance	F*	Significance
Plaque Index	0.188	0.666	5.116	0.026	3.059	0.084
Gingival Index	39.599	<0.001	107.624	<0.001	1.11	0.295
Mean Probing Depth	53.614	<0.001	130.624	<0.001	1.318	0.254
%sites>3mm of Probing Depth	79.85	<0.001	163.004	<0.001	10.204	0.002

*General linear model of repeated measurements.

### Gingival index

The mean value obtained at each measurement increased during the pregnancy [56.7±0.20% at baseline, 66.3±0.17% at 21–23 weeks and 74.5%±0.18 at 34–36 weeks] and decreased [59.3%±0.21] at 3–6 weeks postpartum. Comparisons among the data recorded at the different time points showed a significant difference between the mean at baseline and that at 21–23 weeks and 34–36 weeks (p<0.001). At 3–6 weeks after delivery, the Gingival Index returned to values similar to those at baseline, whereas all remaining comparisons (34–36 weeks vs. 21–23 weeks, and postpartum vs. 34–36 weeks and 21–23 weeks) showed statistically significant differences (Table 1).

### Probing depth

**Fig 1:**
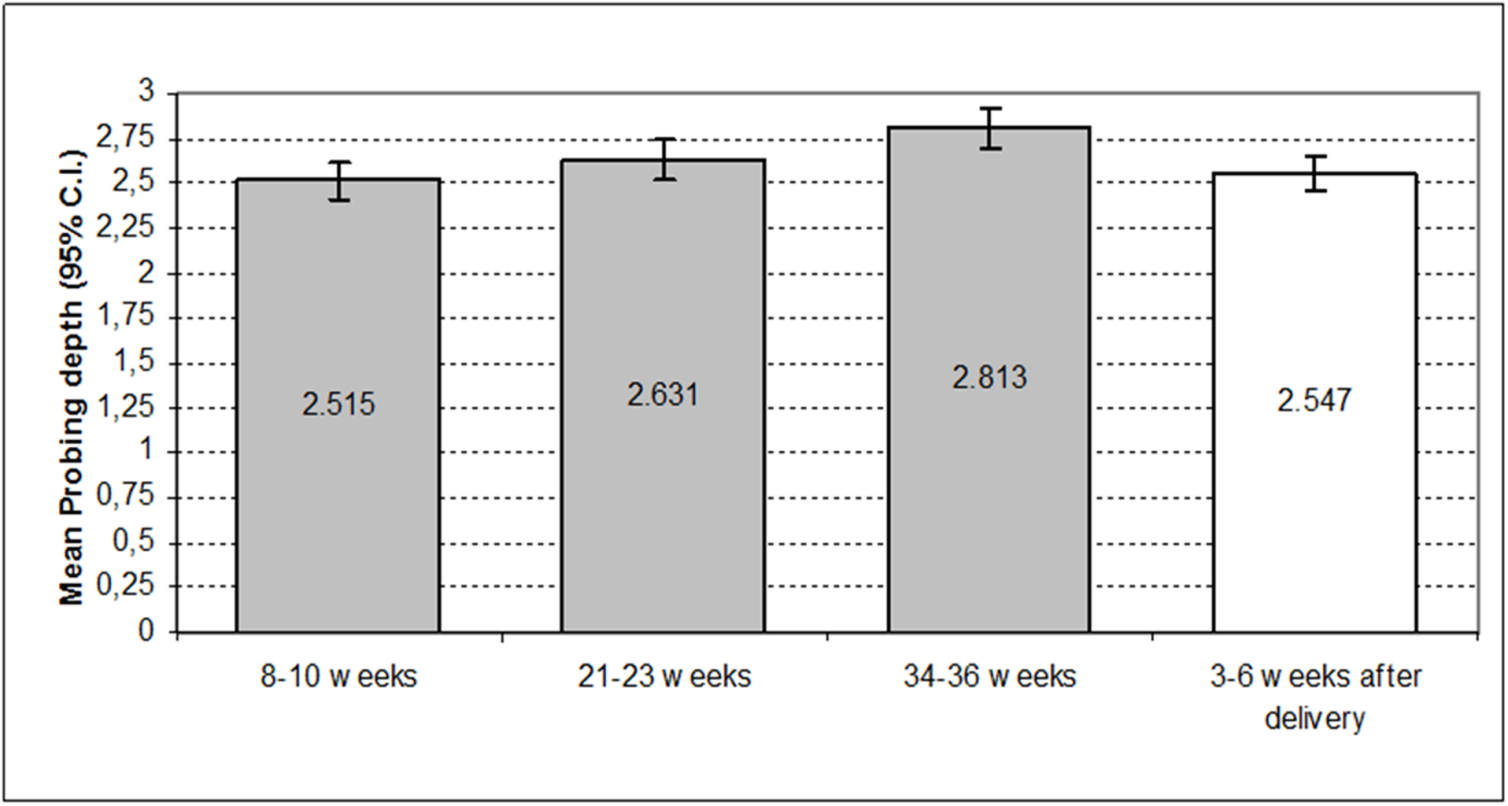
Mean probing depth during pregnancy and postpartum.

Fig 1 show how the mean probing depth in the full-term women increased with the course of their pregnancy. The probing depth significantly increased during the pregnancy (4.6% from baseline to 21–23 weeks, 6.9% from 21–23 weeks to 34–36 weeks) and showed a significant decrease of 10.38% with respect to the measurement at 3–6 weeks after delivery (Greenhouse-Geisser = 71.70; d.f. = 2.11; p<0.001). Table 1 exhibits the results of the single intrasubject comparison test (considering initial baseline measurement as reference category), comparing the probing depth means obtained at each of the four measurements. The mean depth at baseline significantly differed from that at 21–23 weeks and 34–36 weeks (p<0.001). The only non significant difference was between baseline and postpartum values, from 2.51±0.05 to 2.55±0.049.

**Fig 2:**
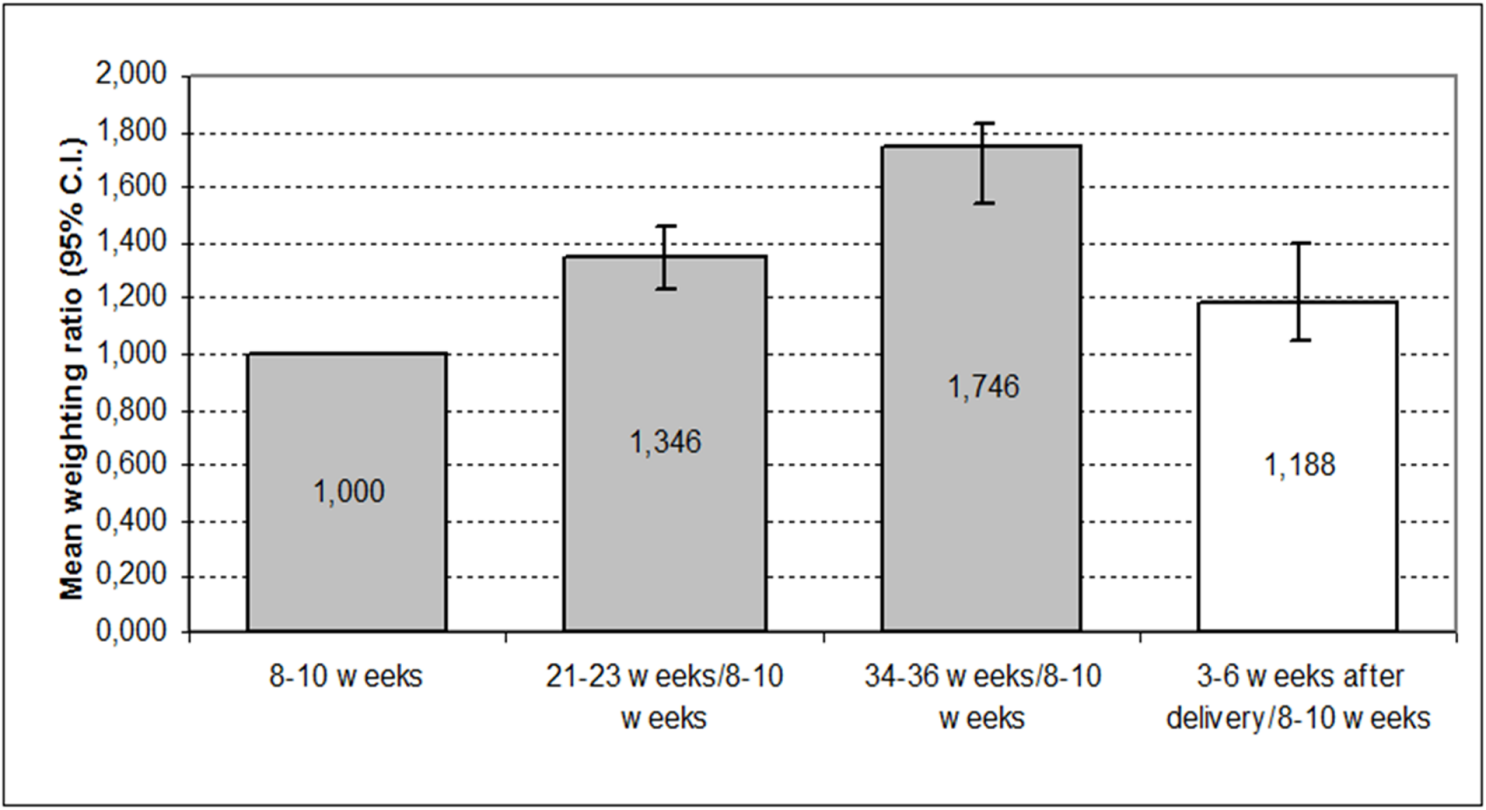
Comparisons of proportion of sites with probing depth >3mm during pregnancy and postpartum.

Fig 2 depicts the proportion of sites with a depth of >3 mm at the different time points, showing a significant increase throughout the pregnancy. The largest change was between baseline and 34–36 weeks with an increase of 74.6%. There was a marked reduction at 3–6 weeks after delivery (47.06%). (Greenhouse-Geisser = 75.31; d.f. = 2.38; p<0.001). The single intrasubject test showed that the baseline measurement significantly differed from those at 21–23 and 34–36 weeks. Furthermore, unlike the other depth measurements, although there was a considerable reduction in the mean percentage of sites at 3–6 weeks after delivery with respect to 21–23 weeks and 34–36 weeks, it remained significantly (p<0.02) higher than the first measurement (Table 1).

## Discussion

Our findings are in agreement with those reported by others authors like Cohen et al. [4], Figuero et al. [21] and Rashidi Maybodi et al. [35], who observed a gradual increase in gingivitis from the first to the third trimester. The improvement during the postpartum was also described in studies by Raber-Durlacher [13] and Abraham-Inpijn et al. [36], or the study of Gürsoy et al. who concludes that changes in clinical parameters during pregnancy are reversible [37]. However, Tilakaratne et al. [38] found no difference in plaque index values between pregnant and non-pregnant women in a study of rural women in Sri-Lanka, although gingival index values significantly increased at each trimester (p<0.01; p<0.001), above all at the third. Three months after the delivery, the levels were similar to those at the first measurement, although the values remained higher than in controls. Unlike the present study, there were no significant differences in probing depth among the trimesters, and the author found gingival alterations, which were likely related to hormonal factors, but no attachment losses. The study by Tilakaratne et al. differed from the present investigation because it was a case-control study comparing 47 primiparus pregnant women (a smaller sample size than ours) with 47 non-pregnant women and it applied different inclusion criteria, excluding patients with any type of periodontal alteration, which was a condition for inclusion in our study. Moreover, the statistical analysis was limited to the calculation of the means and a comparison with an ANOVA test, whereas a general linear model of repeated measurements was constructed in the present study, and rate estimators and error bars were calculated for the evolution of periodontal parameters and the changes produced among the different measurements.

Raber-Durlacher et al. [39] produced experimental gingivitis in a group of pregnant women, allowing the accumulation of plaque and observing that the plaque index was highly similar among the different phases. On the other hand, bleeding on probing was significantly increased during the pregnancy and was always greater than postpartum. Subsequently, the study was repeated in a group of non-pregnant women, observing a much lower severity of the disease. The author concluded that the gingivitis in the pregnant woman was due in part to physiological vascular phenomena induced by increased estrogen and progesterone levels and in part to bacterial plaque. Although the clinical results reported by Raber–Durlacher et al. [39] coincided with the present findings, their study design was very different because they studied nine pregnant women with no periodontal symptoms who were instructed to use no hygiene techniques for two weeks. The same steps were repeated a few months after the delivery in order to compare the results obtained between these situations. In common with our study, they observed that the gingival index and probing depth increased during the pregnancy and recovered after the postpartum. Their sample size and study period were smaller than those of the present study, which followed the pregnant women from the beginning of the pregnancy until 3–6 weeks after delivery.

In an earlier study, Miyazaki et al. [40] assessed 2,424 pregnant and 1,565 non-pregnant women using the CPITN Index. A high percentage of these women presented with some signs of periodontal disease. There were a higher proportion of pregnant women with gingival pockets of >4 mm. This attachment loss increased with the course of the pregnancy with a maximum peak at the 8^th^ month and a reduction to control group levels at the 9^th^. Although their results were obtained using the CPITN Index, they are in agreement with the present findings in confirming the increase in probing depth with the progression of the pregnancy. However, the authors attributed these findings to a gingival enlargement rather than to a loss of attachment, concluding that pregnant women do not require special preventive periodontal programmes. In the present study, the proportion of examined sites with a probing depth >3 mm at 6–8 weeks postpartum remained significantly higher than at the first trimester (Table 1), so that the worsening in probing depth could not be solely attributed to the gingival inflammation induced by pregnancy. Our findings are agree with Rashidi Maybodi et al. [35] who in a longitudinal study concludes that CPITN increased as the month of pregnancy increased, thus, no significant association.

It can be concluded from the present study, in common with other authors like Machuca et al. [24], Soory [41], Mascarenhas et al. [42], or Chung et al. [43] that the periodontal status of pregnant women who already have some periodontal symptoms worsens with the progression of their pregnancy, reflected in the gingival bleeding and periodontal depth findings. Furthermore, although the clinical parameters improved after the delivery and during the puerperium to reach values similar to those measured at the pregnancy diagnosis, the proportion of sites with a probing depth >3 mm remained significantly higher. We therefore believe that periodontal health programmes similar to those proposed by Garbero et al. [32], currently provided by some centres in our national health service, are essential for the prevention and diagnosis of periodontal disease in pregnant women.

## Supporting information

S1 File Study population values. *All baseline atypical values were near clinical normal values without clinical significance
(XLSX)

S2 File Informed consent given to participants. (DOCX)
