# Supplementary material for: Periodontal status during pregnancy and postpartum
Source: PLoS One. 2017 May 19;12(5):e0178234. doi: 10.1371/journal.pone.0178234 (PMC5438174; doi:10.1371/journal.pone.0178234)
Supplement: S2 File — (DOCX) [file pone.0178234.s002.docx]

**DOCUMENTO SOBRE CONSENTIMIENTO INFORMADO.**

**DISTRITO SANITARIO DE LA AXARQUÍA.**

Dña…………………………………………………………………………………………………………….., con D.N.I……………

Manifiesta estar informada de la realización del Proyecto de Investigación, de título **“Análisis clínico de la alteración periodontal durante la gestación y su relación con las constantes neonatales”**, que se desarrolla en el Centro de Salud de Vélez Málaga, dirigido por el Departamento de Periodoncia de la Universidad de Granada.

He sido informada de que participar en dicho proyecto de investigación no supone ningún riesgo para mi salud, ni la de mi futuro hijo, sino más bien todo lo contrario, ya que permitirá conocer posibles problemas de salud y así orientar a su resolución.

Por lo que doy **MI CONSENTIMIENTO** para participar como una unidad de estudio prevista en dicho proyecto, de acuerdo a la información que me ha sido presentada, quedando libre para desistir del mismo en cualquier momento.

Fecha: Firma:
